# Factors affecting job satisfaction and retention of medical laboratory professionals in seven countries of Sub-Saharan Africa

**DOI:** 10.1186/1478-4491-11-38

**Published:** 2013-08-17

**Authors:** Francesco Marinucci, Mtebe Majigo, Matthew Wattleworth, Antonio Damiano Paterniti, Mian Bazle Hossain, Robert Redfield

**Affiliations:** 1 Institute of Human Virology, University of Maryland School of Medicine, Baltimore 21201, USA; 2 Maryland Global Initiatives, Dar es Salaam, Tanzania; 3 School of Community Health and Policy, Morgan State University, Baltimore, MD 21251, USA

**Keywords:** Laboratory professionals, Job satisfaction, Retention, Sub-Saharan Africa

## Abstract

Effective implementation and sustainability of quality laboratory programmes in Sub-Saharan Africa relies on the development of appropriate staff retention strategies. Assessing the factors responsible for job satisfaction and retention is key for tailoring specific interventions aiming at improving the overall impact of health programmes. A survey was developed to assess these factors among 224 laboratorians working in the laboratory programme the University of Maryland implemented in seven Sub-Saharan African countries. Lack of professional development was the major reason for leaving the previous job for 28% of interviewees who changed jobs in the past five years. Professional development/training opportunities was indicated by almost 90% (195/224) of total interviewees as the most important or a very important factor for satisfaction at their current job. Similarly, regular professional development/opportunities for training was the highest rated incentive to remain at their current job by 80% (179/224). Laboratory professionals employed in the private sector were more likely to change jobs than those working in the public sector (*P* = 0.002). The findings were used for developing specific strategies for human resources management, in particular targeting professional development, aiming at improving laboratory professionals within the University of Maryland laboratory programme and hence its long-term sustainability.

## Introduction

One of the major challenges in implementing health programmes in Sub-Saharan Africa is the reliability of medical laboratory services. The diagnostic support of laboratories is essential for a wide range of diseases and testing purposes, both from clinical [1] and public health perspectives [2]. Numerous global initiatives in Africa have focused on clinical laboratory harmonization and standardization [3], and on laboratory accreditation [4]. As a consequence, many programmes over the last decade have been dedicated to building quality laboratory services through training laboratory professionals, upgrading infrastructure at medical facilities, installing new instruments and equipment, and strengthening supply chain systems [5-7]. However, the first barrier for quality improvement at any level of the health care system is human capacity development, which continues to be a gap in implementing health programmes [8-12]. The lack of trained healthcare personnel is widely addressed in numerous programmes [13-15], but often without integrating this training into human resources management at the health facility level.

The quality of medical laboratory operations is driven by technical skills, quality management systems and the motivation of human resources. The technical competency of personnel plays a critical role in ensuring strict adherence to the numerous procedures of the total testing process as defined by the quality management system [16]. To achieve proficiency, laboratory professionals need both targeted training and an appropriate working environment to turn acquired knowledge into technical skills. Numerous efforts have focused on expanding basic coverage of HIV care and treatment, which has resulted in the widespread implementation of new technology throughout Africa. The expanded HIV testing capacity at different levels of the health system, both in terms of the amount of equipment and in technology, requires additional skills for laboratory professionals. Many laboratory programmes that have implemented new technology have not effectively supported the process of developing technical skills with appropriate training and incentives. Due to lack of exposure and incomplete training on new automation, laboratory technicians can see new technology as additional work instead of being able to do more work more efficiently. The laboratory scale-up poses challenges if technologies are implemented without supporting and training laboratory professionals.

The direct repercussion of this is suboptimal service provided to patients. High turnover rates lead to periods of understaffing in the laboratory, creating increased workloads for remaining staff. Overworked laboratory professionals are more likely to ignore Good Laboratory Practice, thereby increasing the number of mistakes and accidents. The magnitude of high turnover rates due to brain drain is not well understood, hence, the need for monitoring health professionals’ movement within and outside specific programmes.

High turnover of laboratory professionals is a drain on programme funds, as more time and resources need to be devoted to advertise, interview, hire, and train new laboratory professionals. High employee-turnover also makes introducing new diagnostics and techniques, research protocols [17,18], quality improvement systems, and policies difficult to implement because new staff lack prerequisite training and do not have the foundation on which to build.

The Institute of Human Virology of the University of Maryland School of Medicine (IHV-UMSOM) assessed the factors affecting satisfaction and incentives for job retention of laboratory professionals at the supported sites in seven African countries. This survey and its results were useful for developing targeted strategies for human resources management aiming at improving laboratory professionals’ retention and therefore, the long-term sustainability of University of Maryland laboratory programmes.

## Methods

Between November 2010 and March 2011, all laboratory professionals working at 229 health facilities supported by IHV-UMSOM in Ethiopia, Kenya, Nigeria, Rwanda, Tanzania, Uganda and Zambia were asked to participate in the survey. For the purpose of this survey, laboratory professionals were defined as anyone working in the laboratory, including: laboratory technicians, laboratory technologists, medical laboratory scientists, laboratory assistants, and microscopists. Based on the level of education the laboratory technologists and medical laboratory scientists were categorized as highly trained laboratory professionals and the others as less-highly trained laboratory professionals.

A 12-question, English-language survey was developed to gather socio-demographic data and to appraise the importance of aspects central to job satisfaction and retention. The factors for inclusion in the survey were selected based on literature review and experience of IHV-UMSOM working and supporting seven countries. There was not much variation in the factors across the countries. For the purpose of this survey, salary was excluded for three main reasons: first, it is not usually reflected as the main factor in staff motivation [19-21]; second, raising salaries of health workers must be sustained by fixing the rise into the complex national pay structure strictly related to country-specific factors [22]; and finally, the funds of the project did not allow IHV to introduce any salary rises.

The choice of having these specific five components for job satisfaction only was dictated by the feasibility of implementing targeted interventions in these areas within the limited timeframe of IHV programme. Laboratory professionals were first asked to rate the factors, important to their current job satisfaction. The five categories covered professional development/training opportunities; benefits (such as health insurance, overtime compensation, cell phone airtime, developmental loan, food/house allowance, and adequate retirement benefits); vacation/time off; working environment/working conditions; and appreciation and recognition from management and/or hospital administration. A five-point rating scale consisted of: most important; very important; not very important; somewhat important; and least important.

The same rating scale was used again to ask participants what incentives would make them most likely to stay at their current job. The five categories of incentives included regular training and professional development; addition of benefit*s* (such as health insurance, overtime compensation, cellphone airtime, developmental loan, food/house allowance, and adequate retirement benefits); increased appreciation and recognition from management and/or hospital administration; increased vacation/paid time off; and laboratory upgrades (such as improved infrastructure and safety, new equipment, and automated technologies).

The survey was administered in English by IHV-UMSOM in-country laboratory teams. Prior to administering the survey, all questions were reviewed and thoroughly explained in English and in the local language when required. The participation in this study was completely voluntary and refusing to participate did not impact laboratory professionals’ position or personal rights at the health facility. There were no direct benefits for the participants. The survey questions and results were completely confidential and personal information, such as name and address, were not collected. This study was cleared by University of Maryland, Baltimore applicable to federal regulation 45 CFR §46.101(2) exempted design.

## Results

### Demographics of laboratory professionals

A total of 257 laboratory professionals completed the survey. Thirty-three incomplete questionnaires were excluded from the analysis. The frequency distribution for 224 laboratorians, according to demographic and work-related variables, is shown in Table 1. A total of 60% (134/224) of participants were male and 40% (90/224) were female. Forty-nine percent (109/224) of the participants had either Laboratory Technologist or Medical Laboratory Scientist degrees and were referred to as highly trained laboratory professionals. Respondents had an average age of 34 years, with the youngest laboratory professional interviewed being 20 years of age and the oldest 64 years of age.

**Table 1:** **Total and sub**-**group frequency distribution by demographic and job**-**related variables**

**Demographic variables**	**Total (n = 224)**	**Less highly trained laboratory technicians (n = 115)**	**Highly trained laboratory technicians (n = 109)**
Gender			
Male	134 (59.8%)	70 (60.9%)	64 (58.7%)
Female	90 (40.2%)	45 (39.1%)	45 (41.3%)
Age group, years			
<25	18 (8.0%)	11 (9.6%)	7 (6.4%)
25 to 29	60 (26.8%)	36 (31.3%)	24 (22.0%)
30 to 34	56 (25.0%)	18 (15.6%)	38 (34.9%)
35 to 39	34 (15.2%)	21 (18.2%)	13 (11.9%)
40 to 44	26 (11.6%)	11 (9.6%)	15 (13.8%)
45 to 49	11 (4.9%)	7 (6.1%)	4 (3.7%)
>50	19 (8.5%)	11(9.6%)	8 (7.3%)
Level of facility			
Health centre	48 (21.4%)	30 (26.1%)	18 (16.5%)
District hospital	96 (42.9%)	51 (44.3%)	45 (41.3%)
Provincial hospital	10 (4.5%)	1 (0.9%)	9 (8.3%)
Regional hospital	24 (10.7%))	12 (10.4%)	12 (11.0%)
Other^a^	46 (20.5%)	21 (18.3%)	25 (22.9%)
Years in professional working experience			
<1	10 (4.4%)	10 (8.7%)	0 (0.0%)
1-2	18 (8.0%)	9 (7.8%)	9 (8.2%)
2-3	36 (16.1%)	19 (16.5%)	17 (15.6%)
3-5	40 (17.9%)	19 (16.5%)	21 (19.3%)
>5	120 (53.6%)	58 (50.5%)	62 (56.9%)
Laboratory jobs held in the past 5 years			
1	96 (42.8%)	53 (46.1%)	43 (39.4%)
2	94 (42.0%)	44 (38.2%)	50 (45.9%)
3	19 (8.5%)	8 (7.0%)	11 (10.1%)
4	5 (2.2%)	4 (3.5%)	1 (0.9%)
5	10 (4.5% )	6 (5.2%)	4 (3.7%)

^a^Faith-based or private laboratories not equivalent to any level.

In terms of professional experience, 46% (104/224) of the participants had less than 5 years of working experience in the medical laboratory field. Twenty-nine percent (66/224) of the laboratory professionals were employed in the public sector, whereas those hired in the private sector, 8% (12/158), 80% (127/158), and 12% (19/158), were employed in non-governmental, faith-based, or private laboratories, respectively: 85% (190/224) of the participants worked in health centre and district hospital laboratories, or equivalent. The remaining 34 laboratory professionals held jobs at provincial or regional hospitals. A total of 57% (128/224) of laboratory professionals switched jobs at least once over the past 5 years, and among those, 90% (115/128) indicated the reason for leaving their last job; only 22% (25/115) said this was due to relocation. The frequency distributions for reasons for leaving the previous job are shown in Table 2, with lack of professional development being the major motive for changing jobs. Male workers were more inclined to change jobs (56%, 75/134), whereas female laboratory professionals (56%, 50/90), were more likely to stay at their current positions.

**Table 2:** **Total and sub**-**group frequency distribution by reasons for leaving last job**

**Reason for leaving last job**	**Total (n = 115)**	**Less highly trained laboratory technicians (n = 57)**	**Highly trained laboratory technicians (n = 58)**
Relocation/left area/family preferences	26 (22.6%)	13 (22.8%)	13 (22.4%)
Excessive/unequal workload	4 (3.5%)	4 (7.0%)	0 (0.0%)
Lack of appreciation/recognition from management	11 (9.6%)	3 (5.3%)	8 (13.8%)
Poor working conditions	15 (13.0%)	10 (17.5%)	5 (8.7%)
Lack of benefits	27 (23.5%)	13 (22.8%)	14 (24.1%)
Lack of professional development	32 (27.8%)	14 (24.6%)	18 (31.0%)

### Rating factors important for job satisfaction

Professional development/opportunities for training were rated highest for job satisfaction by almost 90% (195/224) of interviewees. The second and third highest rated categories were working environment/working conditions and benefits; these were selected as most/very important by 42% (95/224) and 38% (85/224) of the participants respectively. Appreciation and recognition from management and/or hospital administration was the second least selected factor and it was indicated as most/very important by 28% (64/224) of participants. Vacation/time off was rated as most/very important by only 4% (9/224) of laboratory professionals. The rating for job satisfaction factors in the whole sample is shown in Table 3.

**Table 3:** Rating of factors important for job satisfaction

**Rating of job satisfaction factors**	**Professional development**	**Working environment**	**Benefits**	**Appreciation from management**	**Vacation/time off**
Most/very important	195 (87%)	95 (42%)	85 (38%)	64 (28%)	9 (4%)
Not very important	21 (10%)	52 (23%)	61 (27%)	72 (33%)	61 (27%)
Least important/somewhat important	8 (3%)	77 (35%)	78 (35%)	88 (39%)	154 (69%)

N **=** 224 respondents.

### Rating of incentives important for job retention

Among the incentives important for job retention the category that included regular professional development/opportunities for training was the highest rated for staying at a current position by 80% of total interviewees (179/224). The second highest rated category was addition of benefits indicated as most/very important by 44% (99/224), and laboratory upgrades was chosen by 43% (96/224) of the participants.

A total of 27% (60/224) of laboratorians indicated increased appreciation and recognition from management and/or hospital administration as most/very important in keeping them at their current job. The least rated incentive was increased vacation/paid time off, which was selected as most/very important by only 5% (11/224) participants. The rating for job retention incentives for the whole sample is shown in Table 4.

**Table 4:** Rating of incentives important for job retention

**Rating of job retention incentives**	**Professional development**	**Benefits**	**Laboratory upgrades**	**Appreciation from management**	**Vacation/time off**
Most/very important	179 (80%)	99 (44%)	96 (43%)	60 (27%)	11 (5%)
Not very important	27 (12%)	54 (24%)	68 (30%)	68 (30%)	69 (31%)
Least important/somewhat important	18 (8%)	71 (32%)	60 (27%)	96 (43%)	144 (64%)

N = 224 respondents.

Weighted Cohen’s kappa coefficients (κ) were calculated to assess agreement between factors for job satisfaction and incentives for job retention among highly trained and less highly trained laboratory professionals (Tables 5 and 6). The degree of agreement between these factors was important to tailor the interventions for the two groups of participants.

**Table 5:** Agreement and kappa statistics between factors important for job satisfaction and incentives to stay at current job for less highly trained laboratory personnel

**Factors important to job satisfaction**	**Incentives to stay at current job**				
	**Regular professional development**	**Increased appreciation/recognition**	**Addition of benefits**	**Increased vacation/paid time**	**Laboratory upgrades**
Professional development	82.6 (0.28)^*^	-	-	-	-
Appreciation/ recognition	-	85.1 (0.53)^**^	-	-	-
Benefits	-	-	73.9 (0.34)^**^	-	-
Vacation/time off	-	-	-	81.3 (0.55)^**^	
Working environment	-	-	-	-	72.2 (0.42)^**^

Results are presented as % agreement (kappa coefficient).

**Table 6:** **Agreement and kappa statistics between factors important for job satisfaction and** i**ncentives to stay at current job for highly trained laboratory personnel**

**Factors important to job your satisfaction**	**Incentives to stay at current job**				
	**Regular professional development**	**Increased appreciation/recognition**	**Addition of benefits**	**Increased vacation/paid time**	**Laboratory upgrades**
Professional development	77.6 (0.55)^**^	-	-	-	-
Appreciation/ recognition	-	91.3 (0.24)^*^	-	-	-
Benefits	-	-	96.3 (0.31)^**^	-	-
Vacation/time off	-	-	-	72.2 (0.43)^**^	
Working environment	-	-	-	-	71.0 (0.42)^**^

Results are presented as % agreement (kappa coefficient).

The agreement between satisfaction factors and incentives was estimated using the Landis and Koch classification [23], whereby kappa coefficients of 0.21 to 0.40 indicate fair agreement, 0.41 to 0.60 moderate agreement, 0.61 to 0.80 substantial agreement and 0.81 to 1.00 almost perfect agreement. According to this classification, kappa coefficients for agreement between factors for job satisfaction and job incentives for less highly trained laboratory professionals were almost perfect for the categories of professional development, appreciation from management, and vacation/time off. For the categories of benefits and working environment/working conditions, the kappa coefficients were substantial for this group. For highly trained laboratory professionals agreement was almost perfect for the categories appreciation from management, and vacation/time off, and was substantial for the remaining three factors.

## Discussion

In the group over 45 years of age, only 23% (7/30) were female workers, whereas in the age group 25 to 29 years, women represented 55% (33/60) of this specific population. The decrease in number of female workers over time was not affected by the type of health facility nor the title earned, because the distribution of all laboratory professionals was very similar between the two genders across health facilities.

The years of laboratory experience seemed to be an important determinant for changing jobs, with 57% (66/115) of those who changed jobs in the past five years having between two and four years of experience. It was likely that less highly qualified laboratory professionals were more inclined to stay at their current job because their experience and educational level was less marketable and therefore, they had limited employment opportunities. On the other hand, highly trained laboratorians did not change their job as frequently because, most likely, their current job already matched their experience and educational level. In the countries where the survey was carried out, laboratory professionals tend to continue their studies while working, thereby explaining the correspondence between working experience and educational level at the time of relocation.

Laboratory professionals employed in the private sector were more likely to change jobs than those working in the public sector (*P* = 0.002). The lower workforce turnaround found in government facilities was dependent on country-specific factors, mainly salary scale, benefits, and allowances. In some countries the government employment is permanent with accumulated benefits received on retirement, when leaving before retirement results in loss of all benefits. In addition to these elements, an important role was played by the career prospects available in the public compared to the private sector.

Regardless of the satisfaction factors and incentives under consideration, the degree of agreement should be taken into account in the development of corrective actions and policies. As an early warning indicator, policy makers should consider those areas where moderate agreement between satisfaction factors and incentives has been observed. This approach would likely improve the adoption and implementation of national policies at each health facility by tailoring them to the specific findings observed locally.

A limitation of this study was that it did not comprise many laboratories in urban settings, because the majority of the health facilities included in this survey were located in rural and peri-urban areas. At these levels of the health system, demographics and some factors, such as working environment, working conditions and benefits, differed substantially from those present in urban settings and upper level laboratories.

Besides this, factors that might influence the behavior of local labour markets among countries were not considered, and their impact on willingness to seek other jobs should be explored further. In Zambia, laboratory technicians’ salaries in the public sector were more than three times lower than those in the private sector and between 23% and 46% of those paid by non-governmental organizations [24]. This different salary scale probably contributed to the deficiency of laboratory technicians in the public sector in Zambia. In Nigeria it was likely that policies based on rural area incentives of 25% of salary and other benefits contributed to higher staff turnover in the private sector than in the public sector [25]. In addition to government strategies, donors’ interventions also may influence domestic labour markets. In Kenya, where loss of laboratory staff was higher at lower-level facilities [26], gratuity allowances ranged from 12% to 23% from one province to another in the context of the same project [27]. In Tanzania, fluctuations of health sector budget affected allocations to human resources in particular for recruitment, incentives, retention and capacity building [28]. Despite the different strategies adopted to address local needs, national labour markets have similar dynamics due to the chronic problem of understaffed health care facilities. In this scenario it is likely that the mobility of laboratory professionals was not significantly influenced by determinants such as socio-economic factors and educational background.

## Conclusions

This was the first study to assess satisfaction of laboratory professionals within IHV laboratory programmes in seven Sub-Saharan African countries. It contributed to the evidence that specific strategies for human resources management are part of the necessary activities for implementing quality medical laboratory programmes, particularly in areas where new technologies are available for diagnostic purposes (for example, HIV/AIDS treatment programmes).

Based on the data collected, the first type of intervention should focus on the need for training and professional development to bridge this gap. In particular, new approaches for in-service training should be applied to reduce education-related absences from the workplace. Building capacity and training laboratory professionals without disrupting health services is achievable by promoting blended learning techniques aimed at augmenting traditional learning. Blended learning has the advantages of reducing cost and reaching a greater number of students.

A second intervention aimed at improving the retention of laboratory professionals should encompass a more structured strategy for human resource management at health facility level. In-service trainings should be integrated into professional development plans without compromising any incentive other than the proficiency certificate upon completion. The overall goal of retaining laboratory professionals is to improve their competency through the continuous improvement of Good Laboratory Practices in their routine work. Highly motivated staff adhere more strictly to laboratory procedures defined by the Laboratory Quality Management System with the ultimate outcome of improving the quality of medical laboratory services. Strict adherence to diagnostic protocols supports clinical management of patients and also reduces waste of resources. Laboratory professionals who comply with standard operating procedures make fewer errors with lower volumes of invalid and repeated tests.

A third intervention should address gender-specific factors affecting reasons for leaving the job over time. It is therefore important to explore better these factors and develop flexible retention plans accordingly. Without integrating new strategies for laboratory professionals’ retention, the numerous investments in expanding care and treatment will continue to have a substantial drain on resources due to the repetitive re-hiring, and re-training of new staff within the same laboratory.

## Competing interests

The authors declare that they have no competing interests.

## Authors' contributions

FM and MW conceived the study, and designed the overall research project. FM prepared the first draft of this paper. MM and ADP contributed and commented on the draft. MBH participated in the design of the study and performed the statistical analysis. MW commented on drafts. RR commented on drafts and gave final approval of the version to be published. All authors read and approved the final manuscript.
